# Preparation and quality by design assisted (Qb-d) optimization of bioceramic loaded microspheres for periodontal delivery of doxycycline hyclate

**DOI:** 10.1016/j.sjbs.2021.03.046

**Published:** 2021-03-21

**Authors:** Pooja Jain, Abhinav Garg, Uzma Farooq, Amulya K. Panda, Mohd. Aamir Mirza, Ahmed Noureldeen, Hadeer Darwish, Zeenat Iqbal

**Affiliations:** a Department of Pharmaceutics, School of Pharmaceutical Education and Research, Jamia Hamdard, New Delhi, India; b Product Development Cell, National Institute of Immunology, New Delhi, India; c Department of Biology, College of Sciences, Taif University, P.O. Box 11099, Taif 21944, Saudi Arabia; d Department of Biotechnology, College of Sciences, Taif University, P.O. Box 11099, Taif 21944, Saudi Arabia

**Keywords:** Bioceramic, Composite, Quality by design, Central composite design, Microspheres

## Abstract

PLGA (Lactic- co-glycolic acid) coated chitosan microspheres loaded with hydroxyapatite and doxycycline hyclate complex were developed in the present study for periodontal delivery. A modified single emulsion method was adopted for the development of microspheres. Formulation was optimized on the basis of particle size, drug loading and encapsulation efficiency with the central composite design using 2^3^ factorial design. Microspheres were optimized and electron microscopy revealed their spherical shape and porous nature. *In-vitro* study showed initial burst and then sustained release behavior of the formulation for 14 days. Further, *in-vitro* antibacterial study performed on E. coli (ATCC-25922) and S. aureus (ATCC-29213) revealed concentration dependent activity. Also, *in-vitro* cyto-toxicity assessment ensures biocompatibility of the formulation with the fibroblast’s cells. Overall, the quality by design assisted PLGA microspheres, demonstrated the desired attributes and were found suitable for periodontal drug delivery.

## 1 Introduction

Periodontal infections have recently become a significant oral health problem in majority of population all over the world (Eke et al., 2015). Periodontitis comprises a group of diseases affecting the supporting tissue of the teeth often causing inflammation and bleeding of gums (Jain et al., 2020a, Jain et al., 2020b). The pathogenesis of the disease points towards the involvement of bacterial infections, which is mixed in nature (Armitage, 2004). A total of 400 types of microbes have been found to be present in the oral cavity of which the majority constitutes the anaerobic kind such as *Porphyromonas gingivalis, Prevetola intermedia, Fusobacterium* and *Actinobacillus actinomycetemcomitans* (Kilian et al., 2016). These organisms can be easily located at the gingival crevice of the teeth, below the tongue, and in the saliva. They secrete collagenase, which destroys ligament that is a connective tissue of the periodontal membrane, and metabolites from this reaction causes periodontitis (Armitage, 2004). The globally accepted conventional treatment comprises of systemic delivery of large doses of antibiotics, which can kill the pathogens located at distant sites, where dental instruments and topical antiseptics would not reach (Jain et al., 2019). Recently, researchers have proposed films, nano/microfibers, sponges as a drug delivery platform to combat dental diseases such as gingivitis and periodontitis (Baker, 1988). Amongst the afore-mentioned drug delivery platforms, biodegradable microparticles emerges as the most advantageous due to the reason of easy administration and no need of removal after treatment completion as after direct insertion into the periodontal pocket, they can continuously release controlled amount of drug for longer duration (up to a month) (Pragati et al., 2009). The present research is an attempt to develop sustained release periodontal formulation loaded with an antimicrobial agent and a bioceramic. Bioceramic loaded with antimicrobial agent, which shall remain there in and continuously deliver drug in the cavity in concentrations above the MIC(minimum inhibitory concentration) of the antibiotics is an attractive option. The expected outcome of this system is to have minimal toxic effects in the body along with appreciable reduction in the dose. Doxycycline hyclate was selected as an antibiotic as it has low MIC for periodontal pathogens and high half-life in comparison to other antibiotics. It has both the desired potential like antibiotic activity and the ability to block the action of matrix metalloproteinase enzyme (a collagenase) which is responsible for the dissolution of the collagen/connective tissues of the gingiva (Emingil et al., 2004). Moreover, an added advantage would be the combined use of bioceramic/ calcium compounds which would itself cure, promote the bone formation that is usually destroyed /degraded towards the progression of the disease. Bioceramic; hydroxyapatite was employed as the osteo-inductor. And it is the main mineral component of the vertebrate’s skeleton (Okuda et al., 2005). The porous granular HA promotes bone desorption by helping osteoblast cells which are active in bony part. Hence ensure early recovery of periodontal pocket by curing infrabony defects (Victor and Kumar, 2011). In the present research, chitosan microspheres were prepared by emulsion polymerization technique. Apart from antibacterial and antiplaque activity, chitosan promotes osteoblast activity which in turn helps in bone growth (Pang et al., 2005). But chitosan microspheres were not able to provide the long-term sustained release effect, so in order to achieve this PLGA (Lactic-co-glycolic acid) coating was further applied onto the chitosan microspheres. PLGA is biocompatible and biodegradable polymer (Patel and Chakraborty, 2016) and microspheres based on PLGA polymer are very commonly exploited for sustained drug delivery applications (Lu et al., 2000) and also as cell carriers (Ho et al., 2008). Hence the novelty of the current research lies in the fact that the dual agents are incorporated in a singular delivery system of dual polymers and are targeted to combat the periodontitis for longer duration. As process variable have an important impact on the quality of developed formulation, so in the present study, quality by design approach was applied for the development of PLGA microspheres.

## 2 Materials and methods

### 2.1 Materials

The doxycycline hyclate and PLGA 50:50 were obtained as a gift sample from Unicure India and Evonic, Mumbai, India respectively. And Hydroxyapatite was procured from Avanti Laboratories Pvt. Ltd, Hyderabad.

### 2.2 Methods

#### 2.2.1 Fabrication of hydroxyapatite - drug complex

The complex of doxycycline hyclate and hydroxyapatite (1:5 w/w) was prepared by physical adsorption method where the specific amounts of drug and hydroxyapatite were dissolved in 10 ml of water. The solution is sonicated so as to obtain the uniform dispersion. The system was subjected to biological shaker with 25 ± 5 °C temperature and 150 rpm for 12 h. The complexed powder was dried at room temperature and collected. Further this complexed powder was incorporated in the chitosan microspheres.

#### 2.2.2 Fabrication of drug complex loaded chitosan microspheres

Prepared complex powder was suspended in chitosan solution in 1% acetic acid. This aqueous phase was emulsified with small amount of liquid paraffin (oil phase) with prefixed amount of span 80 (0.05%). W/O emulsion was prepared by vortex mixing for 20mins. After emulsification, optimized solution of Na TPP was added to the emulsion and was gently agitated by overhead stirrer at 350 ± 25 rpm and temperature of 40 ± 5 °C. After the cross linking time, the chitosan microspheres were washed with n-hexane repeatedly, filtered and dried at room temperature (Berthold et al., 1996).

In order to obtain an optimized formulation, it was necessary to screen the excipient quantity and experimental conditions. Various formulations were prepared and optimized on the basis of drug: polymer ratio, internal: external volume ratio, rate of stirring, amount of cross-linking agent and duration of cross linking. Effects of these parameters were observed in drug loading and encapsulation efficiency. To optimize each parameter, certain sets of formulations were prepared and the one which was showing the best drug loading and encapsulation efficiency was selected for further characterization. Encapsulation efficiency was calculated with the help of below mention formula (Jain et al., 2020a, Jain et al., 2020b)-

Encapsulationefficiency=(Totaldrug-Amountofunentrappeddrug)×100/Totalamountofdrugaddedintheformulation

#### 2.2.3 Evaluation of optimized formulation

##### 2.2.3.1 Particle size analysis and surface imaging

Particle size was measured by the technique of light scattering using Malvern mastersizer 2000 analyzer. In this technique, a single narrow mode was used within the range of 0.020–2000 µm at high sensitivity.

The sample of optimized chitosan microspheres were suspended in aqueous solvent. A drop of sample was poured on the aluminum sample stage which was dried in an vacuum desiccator for 24 h. The dried sample was sputtered with gold film in the ion-sputtering device (JFC-1100, JEOL Ltd.). Then, the gold coated sample was observed by scanning electron microscope (SEM, JSM-6060LA, and JEOL Ltd.).

##### 2.2.3.2 In-vitro release kinetics

For the *in-vitro* study of optimized chitosan microspheres; 10 mg of formulation was taken in the 5 ml Eppendorf tube and 2 ml of phosphate buffer pH 6.8 (dissolution medium) was added into it. The assembly was then subjected on a biological shaker at temperature of 37 ± 5 °C. 1 ml of sample solution was taken and replaced with 1 ml of buffer solvent at different time intervals of 0, 3, 6, 12, 24, 48, 72 and 96 h. Collected samples were analyzed by UV spectroscopy technique at 288 nm (Jamal et al., 2012).

#### 2.2.4 Fabrication and optimization of PLGA coated chitosan microspheres

A multi-phase W/O/W emulsion or solvent evaporation technique was adopted to developed the chitosan microspheres (Liu et al., 2004). Aqueous solution of chitosan containing doxycycline and hydroxyapatite granules was prepared (called as internal aqueous phase) with span 60 (0.02%) which was emulsified for 20 sec by adding organic phase of methylene chloride solution of PLGA using homogenizer. In this water phase, one drop of PVA solution was added.

The above prepared water in oil emulsion (W/O) was dropwise added (21.5G needle) on the PVA solution (external water phase) using a glass syringe which divided in two phases. In first phase, PVA solution was added to develop dual phase emulsion with continuous stirring for 2.5 h on the magnetic stirrer at constant rpm of 300 ± 25 and temperature 30 ± 5 °C. Rest of the PVA solution was added for methylene chloride removal. Centrifugation of resultant microspheres at 5000 rpm for 20 min was performed, supernatant was discarded followed by washing with distilled water at-least three times to remove traces of solvent from the microspheres and then ultimately filtration was performed. The collected microspheres were dried overnight under vacuum to remove methylene chloride (USP limit = 500 ppm) and stored at 4 °C.

The microspheres were optimized on the basis of drug polymer concentration, oil phase concentration, amount of PVA. Further outer phase volume and speed of stirring was optimized with the central composite design applying response surface methodology of Design Expert software (Design-Expert 11.0.5.0, Stat-Ease, Inc., Minneapolis, United States). As for a nanoparticle formulation encapsulation efficiency, drug loading and particle size all are important parameter, therefore a 2^3^ level factorial design was adopted where independent variables were set as outer phase volume (A) and stirring speed (B) whereas encapsulation efficiency, drug loading and particle size were selected as dependent variables.

#### 2.2.5 Characterization of optimized formulation

##### 2.2.5.1 Surface imaging by electron microscopy (SEM)

The sample of optimized microspheres was suspended in aqueous solvent. A drop of sample was poured on the aluminum sample stage which was dried in vacuum desiccator for 24 h. The dried sample was sputtered with gold film in the ion-sputtering device (JFC-1100, JEOL Ltd.). Then, the topography of gold coated sample (microspheres) was observed by the electron microscope (SEM, JSM-6060LA, and JEOL Ltd.)

##### 2.2.5.2 In-vitro drug release

For the *in-vitro* study of optimized chitosan microspheres; 10 mg of formulation was taken in the 5 ml Eppendorf tube and 2 ml of phosphate buffer pH 6.8 (dissolution medium) was added into it. The assembly was then subjected on a biological shaker at temperature of 37 ± 5 °C. 1 ml of sample solution was taken and replaced with 1 ml of buffer solvent at different time intervals of 24 h up to 14 days. Collected samples were analyzed by UV spectroscopy technique at 288 nm (Jamal et al., 2012). The study was conducted in triplicate manner.

##### 2.2.5.3 In-vitro antimicrobial efficacy study

All the collected samples of *in-vitro* study were deep freezed and subjected to microbiological evaluation. The strains chosen were *E. coli* (ATCC-25922) and *S. aureus* (ATCC-29213) as these microorganisms are implicated in periodontal infections. As per IP or BP standard protocol, agar medium (Mueller Hinton agar) were prepared and kept on the petriplate followed by microorganism plating by the technique of lawn culture. Cups were cut in the media by a sterile borer (borer diameter – 6 mm) and the filtered *in- vitro* release study samples (25 µl) were added to the cups. Doxycyline hyclate solution having a concentration of (5 µg/ml) was used as a control. The above petriplates were placed in the incubator at a temperature of 35–37 °C for the period of 14hrs. After incubation, zone of inhibition were observed and the diameter was measured (Srivastava et al., 2016). All the microbial studies were conducted on the laminar flow hood under aseptic condition.

##### 2.2.5.4 In-vitro cytotoxicity assessment of optimized microspheres

MTT (3-(4, 5-dimethylthiazolyl-2)-2, 5- diphenyltetrazolium bromide) assay was applied to determine the cyto-toxic effect of pure active compound, control and optimized formulation. The assay is based on the fact that, yellow color tetrazolium dye (water soluble) gets converted into purple formazan product (water insoluble) on the living cells. And number of viable cells can be determined by the end product of purple formazan (directly proportional to it). DMEM media containing 10% FBS were used for the growth of NIH-3T3 cells colony (Fibroblast cells) and propagated on the 96 well plates for MTT assay. In duplicates, 20–500 µg/ml concentration of active drug compound, control and optimized formulation were mounted on the 96 well tissue culture plate or Falcon plate. After incubation of 24 h, MTT assay was used to determine the cell viability (Mosmann, 1983). After that, above media solution was discarded by all the wells of plate and 10 µl of MTT reagent was added into each well (Chemicon International, from Millipore) from the stock solution having concentration of 5 mg/ml. The treated plates were placed for incubation at 35–37 °C for 2–3 h, followed by removing the reagents and solubilizing the settled crystals by adding isopropyl-alcohol (IPA). This extract containing Isopropanol and 0.1 N HCl was poured into 96 well-plates. Where HCl was reacted in the culture media leads to change of red color phenol into yellow color without any interference on the determination of MTT formazan. The formazan was dissolved in the presence of Isopropanol to provide blue color homogeneous solution and absorbance was measured (wavelength test solution was 570 nm and reference solution was 630 nm) through the ELISA plate reader.

## 3 Results

### 3.1 Preparation and optimization of composite microspheres of chitosan

The complex of doxycycline and hydroxyapatite was prepared by physical adsorption method. And this, complex was incorporated in the chitosan microspheres. In order to formulate the chitosan microspheres, firstly drug-polymer ratio was optimized. Highly hydrophilic drug had always low entrapment efficiency as compared to the water insoluble drugs. For this reason, both high and low drug to polymer ratio i.e., 2:1, 1:1, 1:2 was considered and three formulations were prepared amongst them the 1:2 batch shows highest encapsulation efficiency.

In the next level, three formulations were prepared to optimize the concentration of cross-linking agents whereas the drug-polymer ratio (1:2) was set fixed. The amount of Na TPP as cross linker was incorporated at three levels as 50–150 mg, wherein the 100 mg was found most suitable. High concentration of cross linker shows high loading capacity and entrapment efficiency and on the same time too much cross linking would lead to rigid structure changes in the chitosan and would affect the mucoadhesive properties of the polymer. Optimum loading capacity and drug encapsulation efficiency was found with 100 mg of cross linker.

Time of cross linking was the optimum parameter for good drug loading and encapsulation efficiency. Sufficient time should be given to the cross linker to form microspheres and to encapsulate drug in the network of polymer chains. Based on this, cross linking time was also optimized at three levels that is 6, 12, 24 h and 12-h time was found to be the most appropriate.

Next to this, stirring rate was optimized at three levels such as 250–450 rpm. The highest encapsulation efficiency was observed when the stirring rate was 350 ± 25 rpm. And F11 was selected as the optimized formulation. Table 1 revealed the constituents of different microspheres and various parameters of characterization of formulations that were optimized during the experiments.

**Table 1:** Optimization of chitosan microspheres with application of formulation variables.

Formulation code	Polymer (mg)	Drug (mg)	Drug: Polymer ratio	HA (mg)	Stirring rate	Cross linker Concentration (mg)	Time of cross linking (h)	Encapsulation efficiency (%)	Drug loading (%) (w/w)
F01	50	100	2:1	0	350 ± 25	100	12	41.3	16.61
F02	50	50	1:1	0	350 ± 25	100	12	58.1	14.53
F03	50	25	1:2	0	350 ± 25	100	12	65.8	9.4
F04	50	25	1:2	100	350 ± 25	100	6	41.48	3.8
F05	50	25	1:2	100	350 ± 25	100	12	77.11	7.01
F06	50	25	1:2	100	350 ± 25	100	24	76.67	6.97
F07	50	25	1:2	100	350 ± 25	50	12	21.35	2.37
F08	50	25	1:2	100	350 ± 25	100	12	77.11	7.01
F09	50	25	1:2	100	350 ± 25	150	12	80.68	6.21
F10	50	25	1:2	100	250 ± 25	100	12	72.31	5.23
F11*	50	25	1:2	100	350 ± 25	100	12	77.11	7.01
F12	50	25	1:2	100	450 ± 25	100	12	27.1	2.46

*F11 is the optimized formulation.

### 3.2 Characterization of chitosan microspheres

#### 3.2.1 Particle size determination and surface imaging

**Fig. 1:**
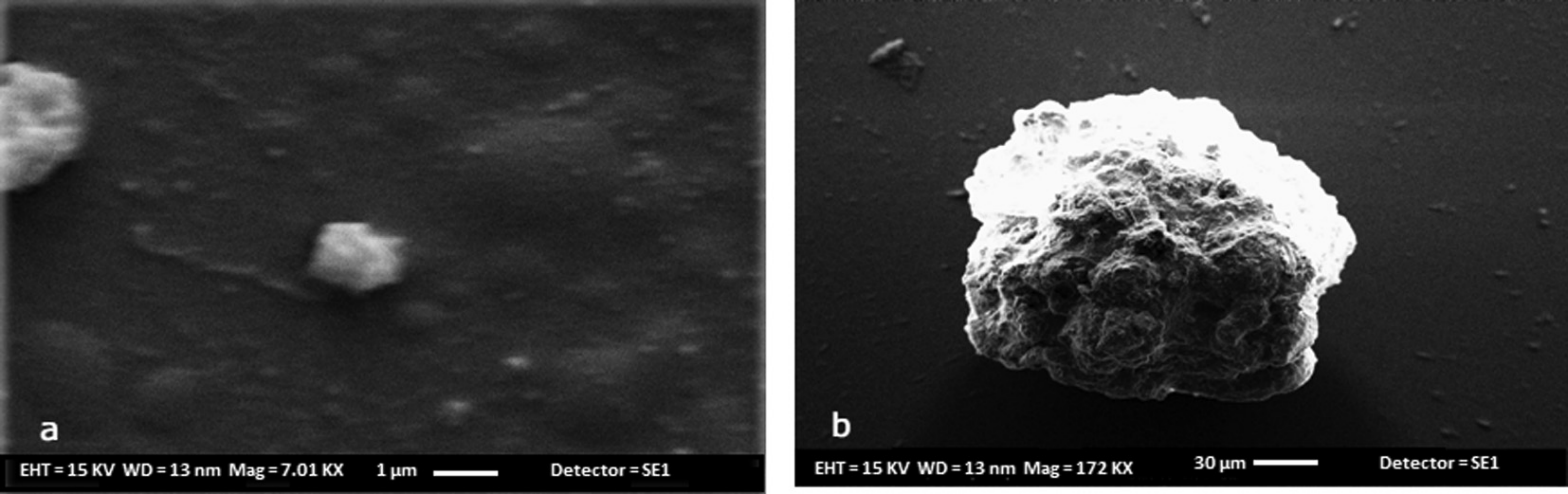
(a) SEM- micrograph of chitosan microspheres loaded with drug and HAP complex, (b) SEM image of isolated chitosan microsphere loaded with drug and HAP complex (isolated microspheres).

The resultant mean particle size of optimized microspheres (F11) was found to be 12 ± 2 µm. Further the chitosan microspheres were fabricated and surface morphology was studied by the SEM imaging. Encapsulation of doxycycline-HAP complex into chitosan yielded micro sizes particles. SEM- micrograph revealed homogenous and spherically shaped particles with small indentations or the rough surface as shown in Fig. 1.

#### 3.2.2 In-vitro drug release study from the chitosan microspheres

**Fig. 2:**
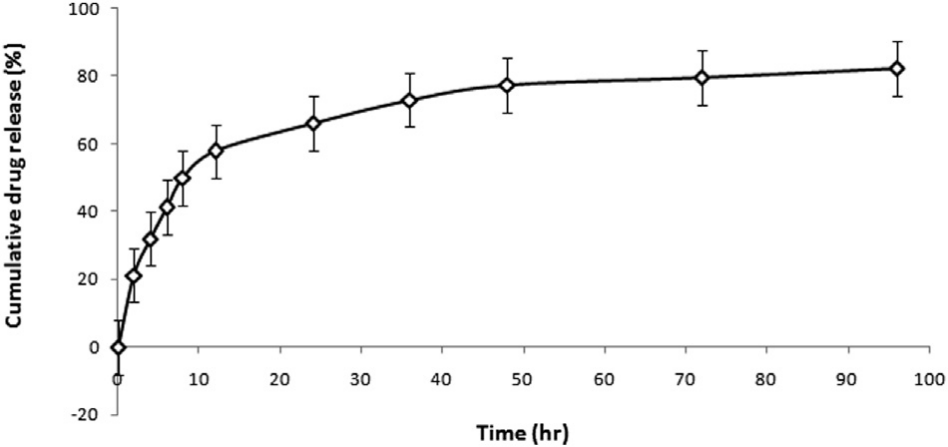
*In-vitro* release of chitosan microspheres depicting cumulative drug release vs time of formulation on the basis of cumulative drug release vs time.

*In-vitro* drug release study was performed in triplicate manner and released profile of the optimized microspheres formulation was studied. These microspheres exhibited biphasic release pattern where the first phase shows the initial burst release of 21.2% as shown in the Fig. 2. Whereas, the second phase shows the sustained drug release up to 4 days.

### 3.3 Preparation and optimization of PLGA coated chitosan microspheres

As the chitosan microspheres alone were not sufficient to provide the sustained release for longer duration of time so further, they were incorporated inside the PLGA microspheres. PLGA microspheres were first optimized on the basis of drug polymer concentration. Table 2 enlists various formulation and their responses for the variables. Preparation of microspheres formulations constitute various ratios of drug-polymer such as 10:1, 5:1, 2:1, 1:1, 1:2 and 1:5. Highest encapsulated efficiency of drug was found in the formulation having 1:5 ratio of drug and polymer. Whereas, the maximum loading capacity was observed in formulation having 1:1 ratio of drug polymer.

**Table 2:** Optimization of PLGA coated chitosan microspheres with application of formulation variables.

Formulation code	Amount of Polymer -PLGA (mg)	Amount of Drug (mg)	Drug: Polymer ratio	HAP amount (mg)	Chitosan amount (mg)	Internal water phase volume (µL)	External oil phase volume (ml)	PVA concentration (% w/v)	Outer phase volume (mL)	Stirring rate (rpm)	Encapsulation efficiency (%)	Drug loading (% w/w)
C01	50	500	10:1	100	5	250	5	2	50	350	69 ± 2.45	47 ± 1.1
C02	50	250	5:1	100	5	250	5	2	50	350	64.5 ± 3.14	34 ± 1.5
C03	50	100	2:1	100	5	250	5	2	50	350	37.31 ± 2.15	3.35 ± 0.23
**C04**	50	50	1:1	100	5	250	5	2	50	350	35 ± 2.37	14.75 ± 2.56
C05	50	25	2:1	100	5	250	5	2	50	350	24.5 ± 2.90	3.5 ± 0.36
**C06**	50	10	1:5	100	5	250	5	2	50	350	78.75 ± 4.01	4.73 ± 0.98
Selected Drug: Polymer ratio = 1:1 & 1:5												
**C07**	50	10	1:5	100	5	250	5	2	50	350	78.75 ± 4.79	4.73 ± 0.9
C08	50	10	1:5	100	5	500	5	2	50	350	63.53 ± 3.87	3.85 ± 0.23
C09	50	10	1:5	100	5	750	5	2	50	350	48.72 ± 3.01	2.96 ± 0.5
**C10**	50	50	1:1	100	5	250	5	2	50	350	35.1 ± 1.67	8.75 ± 1.57
C11	50	50	1:1	100	5	500	5	2	50	350	29.85 ± 0.32	7.69 ± 1.36
C12	50	50	1:1	100	5	750	5	2	50	350	26.56 ± 0.89	6.83 ± 0.38
Selected internal water phase volume **= 250 µl**												
C13	50	10	1:5	100	5	250	2.5	2	50	350	64.85 ± 4.32	3.85 ± 0.37
**C14**	50	10	1:5	100	5	250	5	2	50	350	78.75 ± 5.67	4.73 ± 0.89
C15	50	10	1:5	100	5	250	10	2	50	350	13.65 ± 2.67	0.82 ± 0.12
C16	50	50	1:1	100	5	250	2.5	2	50	350	14.61 ± 2.78	3.56 ± 0.10
**C17**	50	50	1:1	100	5	250	5	2	50	350	35.1 ± 3.23	8.75 ± 0.32
C18	50	50	1:1	100	5	250	10	2	50	350	18.92 ± 1.98	4.87 ± 0.19
Selected external oil phase volume = 5 ml												
C19	50	10	1:5	100	5	250	5	1	50	350	16.45 ± 3.32	1.01 ± 0.1
**C20**	50	10	1:5	100	5	250	5	2	50	350	78.75 ± 4.89	4.73 ± 1.09
C21	50	10	1:5	100	5	250	5	4	50	350	32.21 ± 3.29	2.73 ± 0.78
C22	50	50	1:1	100	5	250	5	1	50	350	19.91 ± 2.39	5.06 ± 1.23
**C23**	50	50	1:1	100	5	250	5	2	50	350	35.1 ± 3.69	8.75 ± 2.47
C24	50	50	1:1	100	5	250	5	4	50	350	33.21 ± 4.81	3.57 ± 0.98
Selected PVA concentration = 2% w/v												
Out of the two, drug: polymer ratio, 1:5 is selected.												

For the optimization of next parameter i.e., volume of internal water phase, preparation of six microspheres formulations containing different volume of water from 250 to 750(µl) and fixing the 1:1 and 1:5 ratios of drug-polymer. Maximum drug encapsulated efficiency and drug loading capacity was attained with internal water phase volume of 250 µl. It was observed that on increasing water phase both drug loading and encapsulation efficiency had been decreased on both the high and low drug: polymer ratio. This inverse rationality can be explained on the basis that when water phase was more, it gave more water front to the hydrophilic drug which provide easy access to the external water phase and lead to low drug loading and encapsulation efficiency. Secondly, improper primary emulsion formation also affected the drug loading capacity and encapsulation efficiency. Furthermore, the optimization of volume of Di-choloromethane (oil), 6 formulations were prepared and oil volume ranged from 2.5 to 10 ml. Maximum loading capacity and encapsulated efficiency was found with the oil volume of 5 ml. When oil phase was in low concentration, it tends to possess high concentration of polymer and oil phase would be more viscous and more difficult to break up in the smaller sizes and further early solvent evaporation and premature solidification of the microspheres. Whereas, high DCM volume affected in the way that it will be remained in the system for longer duration and not allow PLGA to solidify. Also, high concentration wound also leads to crossing of residual limit of solvent.

For the optimization of PVA concentration, six formulation batches were prepared with concentration ranging from 1 to 4%. Maximum drug loading and encapsulation efficiency was observed with the 2% PVA. The presence of high concentration of PVA in the external water phase may increase the viscosity of the double emulsion, resulting in an increased difficulty in breaking up the emulsion into smaller droplets. These microspheres formed had improper surface morphology which was due to the greater residence time of DCM in the PVA system and lead to decreased or minimal drug loading and encapsulation efficiency in the course of 3 h. Whereas, the low concentration of PVA resulted in formation of small droplets which quickly gets solidified. However, decreased residence time leads to fast removal of drug from the internal water phase and resulted in very low drug loading and encapsulation efficiency.

After optimization of PVA concentration in formulation, rest of the process variables were kept constant whereas volume of outer water phase and speed of stirring was optimized with the help of design expert software using response surface methodology. Total 13 formulation batches were prepared with the water volume of 25–75 ml and stirring speed of 200–500 rpm. 2^3^ factorial design was applied and responses studied were encapsulation efficiency, % drug loading and particle size. Table 3 shows the responses of dependent variables. For encapsulation efficiency quadratic model and F-value of 74.15 (p < 0.0500) implies the model is significant. Following mathematical equation describes the relation between particle size and independent factors.

$$Encapsulation\;efficiency=31.18-0.1411*A-1.27*B-0.3650*AB-4.10A^{2}-4.84B^{2}$$

**Table 3:** Observed responses with variable dependent factors.

Formulation Code	Dependent Factor I Outer Phase Volume (ml)	Dependent Factor II Speed of stirring (rpm)	Response I Encapsulation efficiency (%) N ± SD (N = 3)	Response II % Drug Loading N ± SD (N = 3)	Response III Particle size (µm) N ± SD (N = 3)
F01	14.6447	350	23.75 ± 1.23	1.52 ± 2.03	13.43 ± 1.34
F02	75	500	20.23 ± 1.89	1.5 ± 0.65	15.36 ± 1.89
F03	25	500	20.98 ± 0.76	1.07 ± 1.62	13.11 ± 1.31
F04	50	350	31.68 ± 0.54	1.93 ± 1.20	15.02 ± 2.89
F05	50	350	30.34 ± 1.09	1.89 ± 2.01	15 ± 1.78
F06	50	562.132	20.09 ± 1.69	1.07 ± 0.43	13.91 ± 1.67
F07	50	137.868	23.67 ± 2.01	2.57 ± 1.97	16.13 ± 2.43
F08*	50	350	31.2 ± 2.80	2.2 ± 0.34	15.2 ± 0.82
F09	50	350	30.19 ± 1.97	2.3 ± 1.32	15.8 ± 0.58
F10	25	200	22.79 ± 0.34	2.14 ± 1.89	14.68 ± 2.9
F11	85.3553	350	22.98 ± 1.08	2.12 ± 1.46	16.61 ± 1.89
F12	50	350	32.5 ± 2.89	1.82 ± 1.08	14.9 ± 1.82
F13	75	200	23.5 ± 2.30	2.57 ± 1.35	16.93 ± 1.62

*F08 was selected as the optimized formulation.

**Fig. 3:**
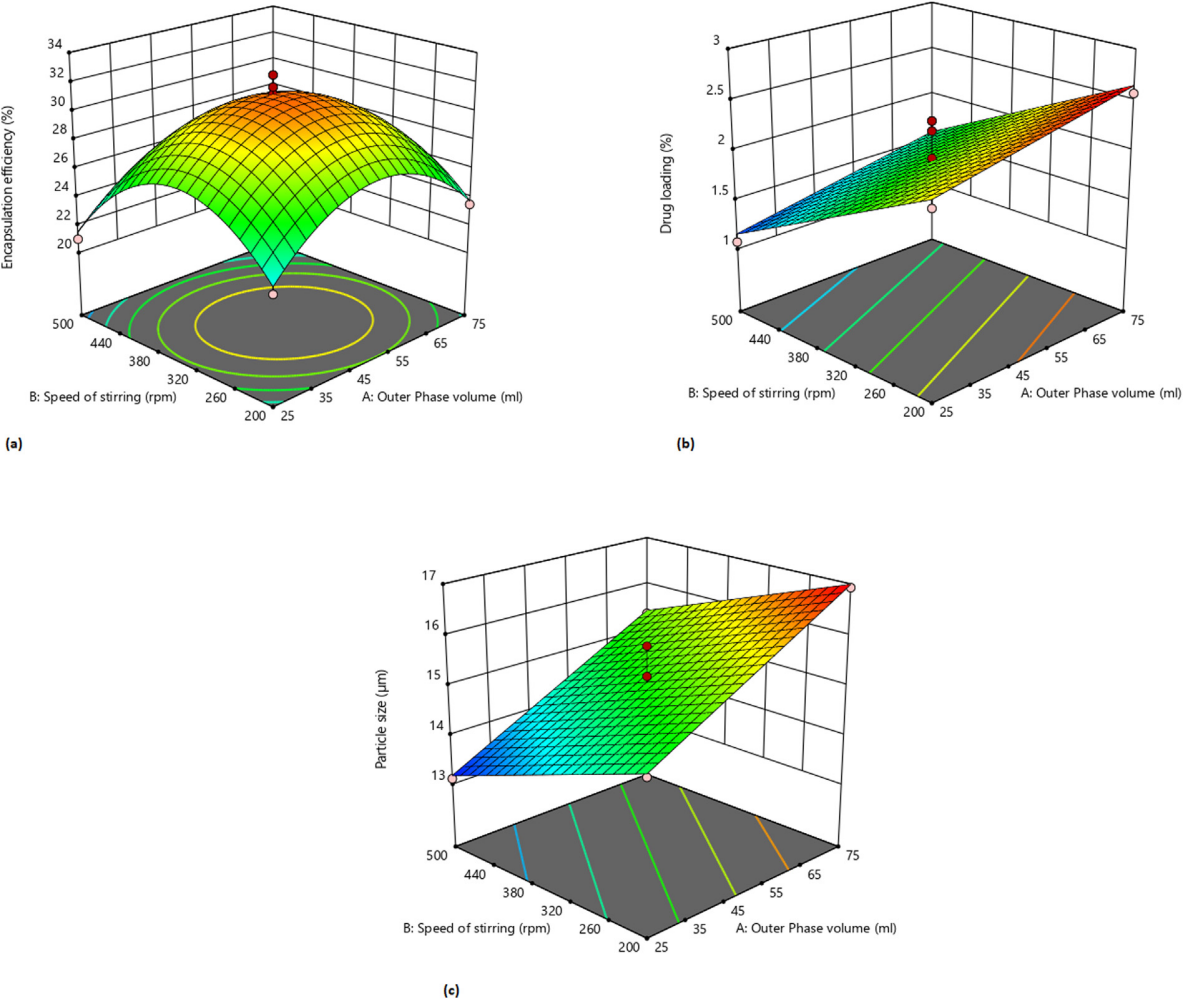
3D Response surface graph showing the effect of outer phase volume and speed of stirring on encapsulation efficiency (a), % drug loading (b) and particle size (c).

ANOVA results indicates that A, B, AB, A^2^, B^2^ are the significant model terms with p < 0.05. This implies that factor A (outer phase volume) B (speed of rotation) and higher order terms have negative impact on encapsulation efficiency. 3D response surface plot in Fig. 3.a depicts the relation between encapsulation efficiency and independent factors.

For drug loading, linear model and F-value of 42.68 (p < 0.0500) implies the model is significant. Following mathematical equation describes the relation between drug loading and independent factors.

DrugLoading=+2.71+0.008∗A-0.00355∗B

ANOVA results indicates that A and B are the significant model terms with p < 0.05. This implies that factor A (outer phase volume) and B (speed of rotation) have positive and negative impact on drug loading respectively. In Fig. 3.b, 3-D response surface plot depicts the relation between the drug loading and independent factors.

For particle size, linear model and F-value of 124.59 (p < 0.0500) implies the model is significant. Following mathematical equation describes the relation between particle size and independent factors.

Particlesize=15.08+1.12∗A-0.7849∗B

ANOVA results indicates that A and B are the significant model terms with p < 0.05. This implies that factor A (outer phase volume) and B (speed of rotation) have positive and negative impact on the particle size respectively. 3-D response surface plot in Fig. 3.c indicates that with low outer phase volume, observed particle size was less whereas with increase in outer phase volume particle size increases. Also, at low stirring speed, observed particle size was high, whereas by increasing the stirring speed, particle size decrease.

### 3.4 Characterization of optimized formulation

#### 3.4.1 Surface imaging of PLGA coated chitosan microspheres by electron microscopy (SEM)

**Fig. 4:**
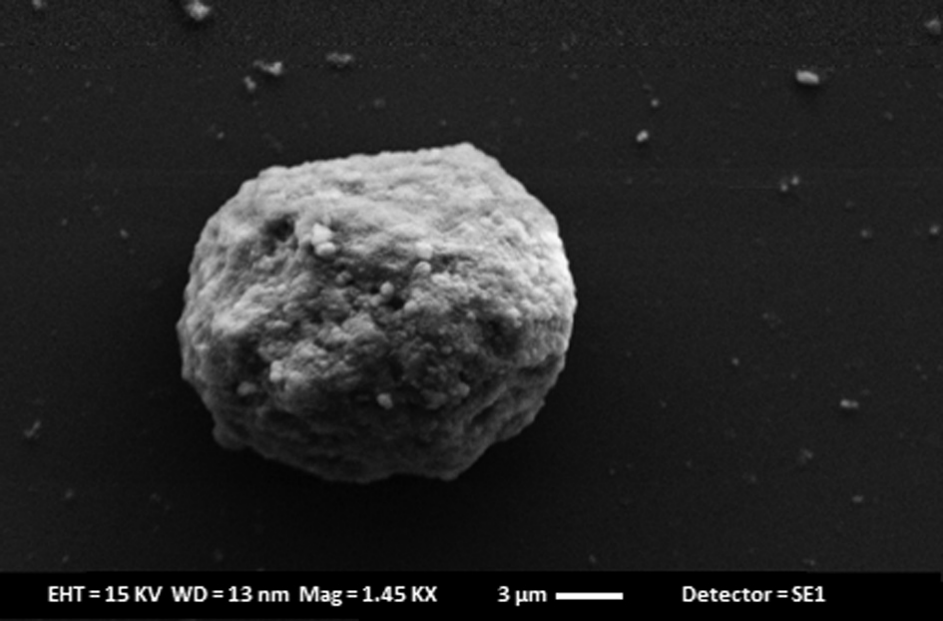
SEM imaging of PLGA coated chitosan microspheres.

To study the surface imaging of PLGA microspheres (F08), SEM was performed and spherically shaped homogenous particles with small indentations or the rough surface was observed as shown in the Fig. 4.

#### 3.4.2 In-vitro drug release study from PLGA coated chitosan microspheres

**Fig. 5:**
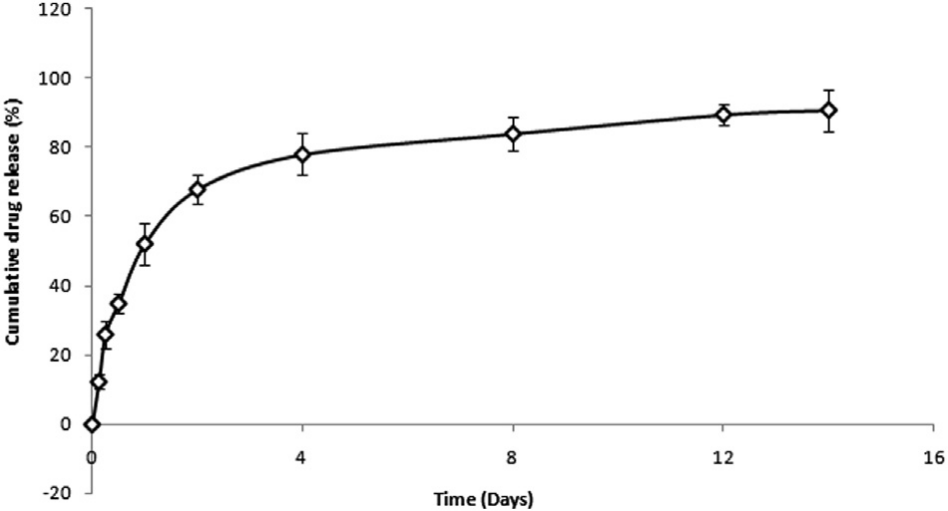
*In-vitro* release of PLGA coated chitosan microspheres depicting cumulative drug release vs time.

For PLGA microspheres, *in-vitro* drug release was performed and samples were analyzed by the UV-spectroscopy. Release pattern follows initial burst phase, where 25.95 ± 0.25% gets released within 6 h. Second phase shows sustained drug release for 14 days as shown in Fig. 5.

#### 3.4.3 In-vitro antimicrobial efficacy study

After conducting antimicrobial efficacy of the formulation samples (as shown in Table 4), it was found that the released samples from the microspheres leads to inhibition of the microbial growth on both the culture mediums of microflora.

**Table 4:** Observed zone of inhibition with the optimized formulation.

S. No.	Time (days)	Diameter of Zone of inhibition (mm) *E. coli* (ATCC-25922) (Mean ± S.D) (n = 3)	Diameter of Zone of inhibition (mm) *S. aureus* (ATCC-29213) (Mean ± S.D) (n = 3)
1	1	28 ± 2.3	34 ± 2.9
2	8	27 ± 1.8	34 ± 2.3
3	12	27 ± 1.9	33 ± 2.2
4	14	25 ± 1.4	33 ± 2.6

#### 3.4.4 In-vitro cyto-toxicity study of optimized microsphere

**Fig. 6:**
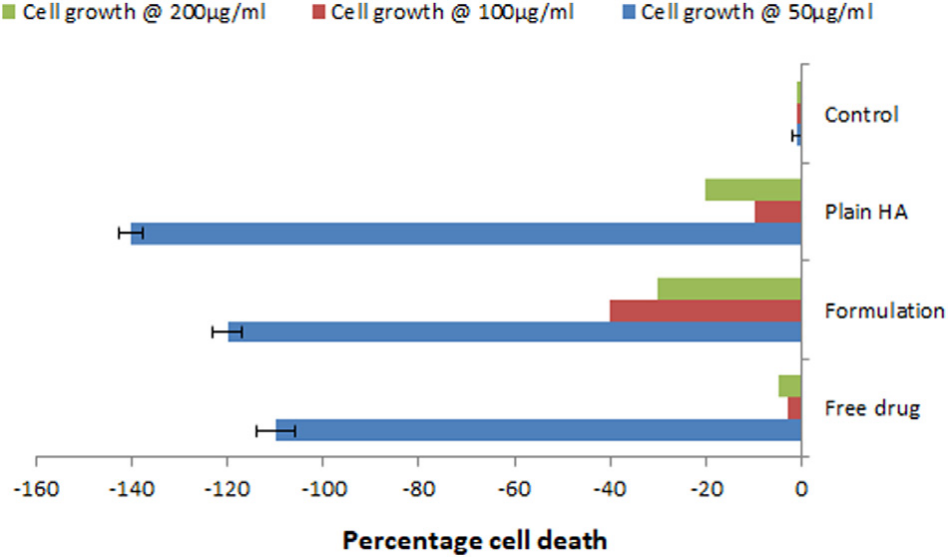
*In-vitro* cytotoxicity assessment of optimized formulation by MTT assay.

Toxic chemicals hamper the normal functions of cells which can be determined by the assessment of cellular damage. In order to assess the biocompatibility of dental materials, Human gingival fibroblasts (Schedle et al., 1998, Kaga et al., 2001) and 3T3 Mouse fibroblasts (Hanks et al., 1991) have been generally applied. These cells show easy extraction from patients, fast growth in culture conditions and enhanced sensitivity in cyto-toxicity study. From the observation of MTT assay (Fig. 6), it was clear that optimized formulation supported the growth of cells at all concentration levels and similar results were observed for plain HA powder and pure drug solutions. Hence it can be concluded that formulation showed no cyto-toxicity and was found to be biocompatible.

## 4 Discussion

The present study was an attempt to develop the PLGA coated chitosan microspheres of HA and doxycycline hyclate complex for periodontal delivery. Although researches have been carried out in the past to develop the PLGA microspheres of doxycycline hyclate but combination of doxycycline hyclate and bioceramic has not yet been tried by the researchers (Hussain et al., 2012, Moura et al., 2015, Ali et al., 2019). So, the novelty of the current work is simultaneous delivery of an antibiotic and a bioceramic through a singular carrier system. For the development of this system, complex (1:5% w/w) of doxycycline hyclate and hydroxyapatite was prepared by physical adsorption method and incorporated into the chitosan microspheres by emulsification method. Various formulation batches were prepared and optimized on the basis of drug: polymer ratio, concentration and time of cross-linking agent and rate of stirring. The effect of these process variables was studied on drug loading and encapsulation efficiency. Results suggests that low drug: polymer ratio i.e. 1:2 gave the highest encapsulation efficiency whereas the high drug: polymer ratio i.e. 2:1 gave highest drug loading. This can be assumed that the at low concentration, polymer was not sufficient to encapsulate all the drug (Hussain et al., 2012). So, in the next level 1:2 ratio was selected for optimization of cross-linker concentration (Na-TPP) and time of cross-linking. Optimum drug loading and encapsulation efficiency was found with the 100 mg of cross linker when the time of cross linking was set for 12 h. With increase in concentration of cross-linker, the molecular distance between the cross-links decreases and hence the entrapment of drugs decreases inside the microspheres (Hussain et al., 2012). Also speed of stirring is an important parameter to affect the drug loading and encapsulation efficiency. And 350 ± 25 rpm stirring rate was found to be the most optimum. High stirring rate had greatly affected the loading capacity of drug and entrapment efficiency. There might be a possibility that too much shearing gave the drug particle to be suspending in the system and not encapsulated in the polymer matrix however when stirring rate was very less, microspheres were not formed as precipitation of polymer-cross linker system had taken place. Particle sizing and scanning electron microscopy of the optimized microspheres depicts the size of 12 ± 2 µm and homogeneous rough surface respectively. Roughness on the surface might be due to the activity of hydroxyapatite. Size of the microspheres is an important parameter to influence the encapsulation efficiency and *in-vivo* biodistribution (Han et al., 2016). And in case of periodontitis, smaller spheres are more preferable as they can easily penetrate the periodontal tissue (Kashi et al., 2012). Also, particle size is important in determining the degradation and release behavior of the microspheres (Ramazani et al., 2015). Biphasic release response with initial burst release and later sustained behavior for four days was observed. Inside the microspheres, Na-TPP-chitosan layer was acting as a diffusion barrier which limits the drug release. This rate limiting barrier was affected by the hydroxyapatite which would be present on the surface and resulting in greater rate of diffusion across the membrane. Also when hydrophilic drugs such as doxycycline hyclate is incorporated inside the chitosan microspheres, the rate of release is expected to be rapid (He et al., 1999).

As the chitosan microspheres alone were not sufficient to provide the sustained release for longer duration of time so further, they were incorporated inside the PLGA microspheres. PLGA microspheres were optimized for drug: polymer ratio (% w/w) and it was found that maximum encapsulation efficiency and drug loading was obtained with 1:5 and 1:1 respectively. This might be due to the aqueous solubility of doxycycline hyclate. Further, PLGA microspheres were optimized with central composite design using Design expert software. A 2^3^ factorial design was applied with volume of outer phase and speed of stirring as independent variables whereas, encapsulation efficiency, % drug loading and particle size were studied as the dependent variables. Out of the 13 formulation batches, F08 formulation batch was selected and optimized formulation was characterized for surface morphology. SEM analyses showed spherical shape, homogeneously rough surface particles or small indentations which might be due to the autocatalysis of PLGA polymer by its carboxylic end group. This autocatalysis will promote the faster biodegradation of the polymer, hence microspheres (Zolnik and Burgess, 2007). *In-vitro* release study revealed an initial burst with a sustained release for 14 days. The sustained release might be attributed to the mechanism of diffusion or erosion which is followed by the PLGA matrix (Han et al., 2016). By comparing the values of diameter of zone of inhibition in, *in-vitro* antibacterial study for bacteria such as *Escherichia coli* and *Staphylococcus aureus,* it can be assumed that drug concentration above the MIC were present in the release samples of 14 days as noticeable diameter of inhibition were observed. *In-vitro* cyto-toxicity study revealed that the plain hydroxyapatite, pure drug and optimized formulations at all concentrations supports the growth of fibroblasts cells (NIH-3T3 cells). However maximum cell growth is observed with hydroxyapatite at 50 µg/ml. Based on the above findings it can be concluded that the PLGA coating on the chitosan microspheres helps to provide the sustained drug release behavior for longer duration and minimal cell toxicity hence making this carrier system as a suitable candidate for periodontal drug delivery.

## 5 Conclusion

It can be concluded that in the present research work quality by design approach was successfully applied to develop the PLGA coated chitosan microspheres of HA and doxycycline complex. The sustained effect of the formulation was attributed by the coating of PLGA. From the *in-vitro* efficacy studies it could be expected that the above-mentioned formulation will give promising results in clinical isolates. Hence, the proposed delivery system with optimum dose and sustained effect would be a better alternative to the high dose conventional dosage forms.

## 6 Summary points

1. The study is based on the development of sustained release periodontal formulation loaded with an antimicrobial agent and a bioceramic.
2. PLGA coated chitosan microspheres were developed and optimized for periodontal drug delivery.
3. The novelty resides in the fact that, in a singular drug delivery system, two therapeutic agents; doxycycline and hydroxyapatite were loaded.
4. A complex of doxycycline hyclate and hydroxyapatite (1:5% w/w) was prepared by physical adsorption method.
5. Chitosan microspheres containing complex, were prepared by emulsification method, optimized and further PLGA coated chitosan microspheres were optimized on the basis of Qb-D approach.
6. PLGA coating was done on chitosan microspheres to attain the drug release for longer duration and the observed in-vitro drug release was upto 14 days whereas with alone chitosan microspheres, 4 days drug release was the maximum.
7. *In-vitro* antibacterial study conducted on *E. coli* (ATCC-25922) and S. aureus (ATCC-29213) indicates that the drug concentration above the MIC was maintained in all the release aliquots.
8. *In-vitro* cyto-toxicity study revealed that the plain hydroxyapatite, pure drug and optimized formulations at all concentrations supports the growth of fibroblasts cells (NIH-3T3 cells), hence are cyto-compatible in nature.

## 7 Ethics approval

Not Applicable.

## 8 Consent to participate

All authors consent to participate in this manuscript.

## 9 Consent for publication

All authors consent to publish this manuscript in Saudi Journal of Biological Science.

## 10 Availability of data and material

Data will be available on request to corresponding or first author.

## 11 Code availability

Not Applicable.

## Declaration of Competing Interest

The authors declare that they have no known competing financial interests or personal relationships that could have appeared to influence the work reported in this paper.
